# 2D Material‐Based Photodetectors for Infrared Imaging

**DOI:** 10.1002/smsc.202100051

**Published:** 2021-10-18

**Authors:** Zhongzhou Cheng, Tong Zhao, Haibo Zeng

**Affiliations:** ^1^ MIIT Key Laboratory of Advanced Display Materials and Devices Institute of Optoelectronics & Nanomaterials College of Material Science and Engineering Nanjing University of Science and Technology Nanjing 210094 China

**Keywords:** 2D materials, infrared detection and imaging, photodetectors

## Abstract

As a dynamic research field, infrared (IR) detection and imaging presents broad prospect in spectra‐chemistry, biomedicine, and artificial intelligence. Due to the excellent properties, 2D materials are expected to overcome the serious manufacturing cost and integration problems of Si‐based photodetectors, providing opportunities for the universal application of IR imaging. Up to now, dozens of 2D materials are used to manufacture IR photodetectors. The ultrathin and flexible 2D material‐based optoelectronic devices, which are easy to machine and integrate, will demonstrate the application beyond imagination. In this review, first, the principle of IR detection and imaging is introduced, in which the single pixel and array imaging are discussed. Then the promising materials, toward broadband detection and special detection, are introduced in brief and the devices for imaging are summarized, including the mode based on light intensity and the strategy originated from polarization. Finally, it is prospected that the 2D materials have the capability to show the integral IR world, which contains large amount of valuable information such as molecular vibration spectra and significant applications such as artificial retina.

## Graphical abstract

2D materials are expected to overcome the serious manufacturing cost and integration problems of Si‐based photodetectors. The ultrathin and flexible 2D material‐based optoelectronic devices will demonstrate the application of infrared (IR) imaging beyond imagination. This review introduces the principle of IR detection and imaging. The promising materials and devices for IR detection and imaging are listed and discussed.

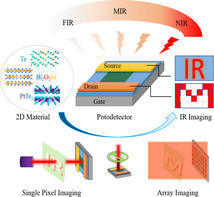

## 1 Introduction

Light carries information, the more light that can be seen, the more world we have. From black‐and‐white image depend on whether the optical signal can be detected, to grayscale image by obtaining the intensity and colorful image by distinguishing the wavelength, even to quantum image from the spin or polarization information, the light detection, and imaging show the important phenomena and processes of the real world.^[^ ^1^ ^]^ Especially, the infrared (IR) spectra contain much characteristic information about the molecular vibration and thermal radiation, which is of great significance for scientific research and industrial application.^[^ ^2^ ^]^ The detection of IR can be used for molecular chemistry, military medicine, spectral analysis, biomedical imaging, artificial retina, environmental monitoring, IR communication, and optical interconnection application, etc.^[^ ^3^ ^]^ It is urgent to develop high‐performance and high‐integrated IR photoelectric detection and imaging devices, in which the materials play a key role. The research on human eyes shows that two kinds of information are formed in the process of seeing the visible light. The one is based on the response to different intensities of the light to become light and shade contrast, and the other is based on the response to different wavelengths to become color contrast. Therefore, we can see the object as color blocks of light and shade. IR imaging can transcend the perception of human eyes, with the broadband detectors feeling the light and shade contrast to generate geometric structure information, and the special‐band detectors sensing colors to obtain physical properties of the objects. For example, the thermal radiation spectra can be collected to form hotspot imaging, which is mapping the regions with different temperatures on the surface of the objects. In addition, some information that human eyes cannot recognize, such as polarization and phase position of a light, can also be caught using special photodetectors, which will absolutely surpass human eyes in another dimension.

IR is an electromagnetic wave between visible light and microwave. According to the different wavelengths, IR can be divided into near IR (NIR, 0.8–2 μm), mid‐infrared (MIR) (2–25 μm), and far IR (FIR, beyond 25 μm) in brief. More detailed division is various depended on the special application, such as terahertz wave (THz, 30–300 μm). The photon energy of IR is from several meV to little eV. The photon energy of near IR is larger (above 0.5 eV) than that of MIR (0.05–0.5 eV) and FIR (below 50 meV). The lowest intensity can be a single photon. Moreover, the main peak of black body radiation is about 10 μm (0.12 eV) at room temperature (298 K), which belongs to the MIR. And the difference between vibrational energy levels in molecules is 0.05–1 eV, and the corresponding photon wavelength is about 1–25 μm. The absorption and emission spectra of the transitions by molecular vibration are just located in the NIR and MIR regions. Therefore, IR detection has a large background noise, which poses a serious challenge to improve the detectivity and responsivity of the detector and the anti‐interference capability.^[^ ^4^ ^]^

The discovery of 2D materials with atomic thickness provides a strong support for the IR detection and imaging. More than 5000 kinds of 2D materials, including near 2000 kinds of layered compounds, with the band gap from 0 to several eV, make the 2D‐based photodetectors have broad prospects.^[^ ^5^ ^]^ And needless to say the countless doping, heterojunction and other modification and assembly way to change the properties of the 2D materials.^[^ ^6^ ^]^ Experiments show that a large number of 2D materials can be used for IR detection and imaging, such as V–VI semiconductors an VIII–VI semiconductors, with the responsivity and detectivity up to 10^8 ^A W^−1^ and 10^15^ Jones, respectively. Huge amount of high‐performance materials gradually constitutes the hardware equipment of IR detection, which can be used in the wide spectrum imaging based on intensity and polarization, opening the industrial applications of night vision, temperature monitoring, and medical imaging. At present, the largest part of the market of photodetectors is provided by the silicon‐based semiconductors, with high performance, high maturity, and high integration of electronic devices. The IR detection that outside the bandgap of silicon depends on the external semiconductors such as InGaAs and HgCdTe systems. Although the materials provide high performance, they have serious manufacturing cost and integration problems, also with rigid and fragile. Therefore, the traditional photodetectors are limited to small batch and high value markets, which has a great block on the application of IR detection and imaging.

This huge challenge provides opportunities for development of 2D material photodetectors, bringing significant market value and profound sci‐economic impact. The advantages of 2D materials in structure and performance can easily form small, flexible and transparent devices. In addition, 2D materials are easy to synthesize with a wide variety, which can be stacked, doped and micro‐nanoprocessed arbitrarily, resulting in a large number of new physical and chemical properties.^[^ ^7^ ^]^ Thus, the 2D materials show many unique characteristics. Especially in the field of photodetection, the adjustable bandgap of 2D materials can absorb a wide spectrum. High carrier mobility and heat transfer efficiency bring about high sensitivity detection. The 2D materials, with the thickness of nano or subnanometer, can form ultrathin devices, which is convenient for the integration of physical and chemical properties. The developed micro‐nano fabrication technologies can be used for artificial atom assembly and segmentation, to produce more magical properties. The production process is compatible with silicon and easy to integrate with mature semiconductor technology. Compared with the latest thin‐film technologies such as InAs, InSb, GaAs‐InGaAs quantum wells, and silicon alloys, 2D materials are unnecessity of high‐vacuum materials growth technique, and are suitable for seamless integration in various substrates (rigid or flexible, single crystal, or amorphous, etc.), so as to realize the integration of high‐performance optoelectronic devices with ultralow weight, high density, and flexibility.^[^ ^8^ ^]^ The 2D material with compact structure and various devices, compatible to Si and easy to machine and integrate, the flexible, and transparent characteristics to fold, will be further stimulated the imagination of the application.^[^ ^9^ ^]^

**Figure 1:**
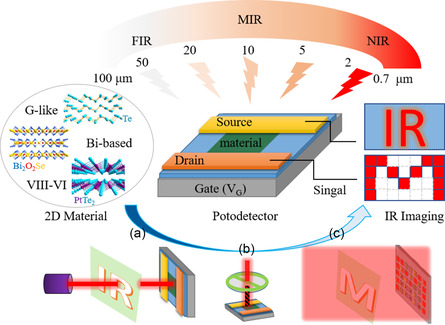
The scheme of IR detection and imaging based on 2D materials. Left: the typical 2D materials of G‐like, Bi‐based, and Group VIII–VI semiconductors with the structure of Te, Bi_2_O_2_Se, and PtTe_2_. Middle: Schematic diagram of a photodetector with the IR illumination from 0.7 to 100 μm upon. Right: The IR imaging based on single pixel and array mode. Down: the mechanism of a) single pixel imaging, b) polarization detection, and c) array imaging.

Combined with the unique properties of 2D materials, and advanced characterization techniques covering the entire process from material growth to device preparation and to performance analysis,^[^ ^10^ ^]^ we believe that 2D‐based IR photoelectric detection and imaging have a wide range of significance in the science. For example, it will be used in the vision of invisible world. And the deeper development of 2D material optoelectronic devices, the wider application of night vision, meteorology, small biomedical, etc. Therefore, it is necessary to conclude the progress of IR detection and imaging based on 2D material in recent years. With the reviewing and summarizing, as shown in **Figure** **1**, we will show some new scientific discoveries and technologies, to forecast the future of the IR imaging.

This review main discusses the material selection and device design for IR imaging, to explore the most advanced detection and imaging scheme for potential application. First, we introduce the principle of IR detection in brief. And then we discuss the imaging strategy based on the information obtained by the detection, including single pixel imaging and array imaging. Then, the materials that can be used in IR detection and imaging are listed one by one, including broadband detection materials and structures, such as V–VI semiconductors and van der Waals heterojunction, and some special‐band detection such as for MIR. It hopes to give an overview of the material selection in IR detection field. Based on the information obtained by the detection, the designs of devices in practical experiment for IR imaging are summarized, which include the imaging based on the intensity, wavelength, and polarization information of the light, respectively. And the integrated designs of the devices are also mentioned and prospected. Finally, the possibility of simulating and expanding human eyes is put forward to explore the future of displaying an integral IR world based on integrated detection imaging. They may contain the valuable information for research and great value for application.

## 2 Principle of IR Detection and Imaging

The photodetector is to convert the received light signal into the electrical signal. Among them, light absorption is the basis, and the change of electrical properties in materials after absorbing light will causes the change of electrical parameters such as current and voltage in the detection circuit, which is convenient to be observed. The essence of IR radiation is the energy release process of electrons, atoms or molecules, which is from the excited state with higher energy level to the lower energy level. The emitted energy is the difference between the two energy levels, shown as a photon with a specific frequency. Photoelectric detection is faced to application, and some specific indicators should be concerned in application. There are three most important detection indexes to evaluate the photodetectors, including responsivity (*R*), response time (*T*), and specific detectivity (D*). The responsivity (or sensitivity) is characterized by the output signal (such as light current or potential) per radiant power, with the unit of A W^−1^ or V W^−1^. The response time include rise time and fall time, which are defined as the output level rising or decreasing from 10% to 90% of the peak, respectively. The specific detectivity (D*) is the reciprocal of noise equivalent power (NEP), which is the radiant power as the noise is equal to the output signal, normalized by per square root of the area and frequency bandwidth. In addition, the 5S requirement of sensitivity,^[^ ^11^ ^]^ high signal‐to‐noise ratio, high spectral selectivity, high Speed, and high stability, also is used to evaluate the performance of a photodetector. For imaging applications,^[^ ^12^ ^]^ high sensitivity is needed for clear imaging and short response time is needed for high frequency modulation.

### 2.1 IR Detection

**Figure 2:**
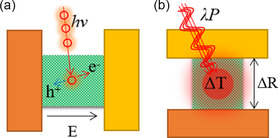
The principle of a) type‐PE and b) type‐PT for IR detection.

At present, a large number of literatures have discussed the main mechanisms of photoelectric detection in detail. According to the way of material absorbing photons, the detector can be divided into photoelectron transition type (type‐PE) and photothermal effect type (type‐PT), as shown in **Figure** **2**. The type‐PE is that photons cause the bandgap transition of electrons in the material to become a built‐in electric field (*E*), changing the number of carriers and the electrical properties of the material. According to the different electrical parameters measured, it can be divided into photovoltaic effect (PVE), photoconductivity effect (PCE), and photogating effect (PGE). The type‐PT is that the incident wave (*λ*, *P*) cause the local temperature rise of the material (Δ*T*), which causes the change of carrier migration rate, resulting in altering the electrical parameters of the material (Δ*R*). The type‐PT includes pure radiative thermal effect (RTE) and photothermoelectric effect (PTE). The photon energy of NIR to MIR is high, which can be caught by the type‐PE photodetectors. While the MIR to FIR is usually detected with a low‐temperature equipment for cooling to eliminate the thermal radiation interference by the detector itself.

#### 2.1.1 NIR to MIR

When the photon energy is larger than the bandgap, the electrons and holes separate from the absorbed photons and form the built‐in electric field, or generate the short‐circuit current or open‐circuit voltage, which leads to the difference of light and dark signals. It is characterized by low dark current, high photon–electron conversion efficiency (quantum efficiency). The PVE photodetectors can work under zero bias. The principle of construction is to separate electrons and holes in space, such as photodiodes formed by p–n junction or Schottky junction. PCE is though photogenerated carriers to strongly change the material resistance, resulting in a strong contrast between photocurrent and dark current. The advantage is that a single piece of 2D material can be added with an electrode without additional processing. The disadvantage is that it needs to work under bias voltage, such as the photoconductive transistor formed by common 2D materials. When the electron or hole in the material is in the trap state, the charged trap state can be used as a local floating gate to strongly regulate the channel conductance, resulting in the difference between photocurrent and dark current. This is the PGE, which is a special case of PCE. It has the advantage of high *R* and the disadvantage of long *T*. The role of traps in the photocurrent generation mechanism in thin InSe photodetectors has been reported.^[^ ^13^ ^]^ And according to the PGE, we can obtain avalanche photodetectors with ultrahigh gain.^[^ ^14^ ^]^ The aforementioned is the photoelectric detection generated by transition absorption, which can be fine tuned to improve the performance of the photodetectors.

#### 2.1.2 Beyond MIR

For detection of MIR to FIR, there is large environmental interference and low energy of the photon. It is difficult to use the type‐PE photodetectors to obtain the single, but it can use the thermal effect to detect. It is necessary to build a stable and highly sensitive energy channel for accurate detection and special imaging. PTE is from the thermal effect caused by the absorption of photons by phonons, which leads to the different temperatures in the material. The change of the temperature causes the different movement of the electrons, leading to the generation of potential difference. The characteristic of the voltage produce by the PTE (*V*_PTE_) is that it does not need bias voltage, but the value of the V_PTE_ is very small. Usually, the *V*_PTE_ is between tens of μV and tens of mV, which requires high‐quality ohmic contact between the electrode and the detection material to obtain larger gain. After the photothermal effect, the resistivity of the material changes with the temperature, and the current through the material also changes, which leads to the difference of light and dark signals. Photothermal detectors do not rely on photoelectric effect, and have no requirements for the matching between the photon energy and bandgap of the materials. The detection range is wide, but the phonon absorption is usually far slower than the transition absorption. The response time of photothermal detectors will be longer.

In addition, there are always other mechanisms to be found out for IR photodetectors. The electromagnetic induction well (EIW) is generated in a metal–semiconductor–metal (MSM) structure, with the metal as the wall of the well and the semiconductor as the bottom of the well. The size of the well usually is less than the wavelength of light. As the photo incidence in the well, the electrons in the metal will be stimulated and injected in the well captured by the semiconductor. Thus, the conductivity of the semiconductor is changed and the optical voltage signal is collected between two metal contacts. The mechanism of EIW is very suitable for long wave radiation because it is not based on the photoelectric effect and there is no need to match the photon energy with semiconductor bandgap. This mechanism has been successfully applied to FIR detection of 2D material photodetectors.^[^ ^15^ ^]^ In addition, according to the record‐low heat capacity and sharp superconducting of the magic‐angle bilayer G (MAG), the single photons can be detected by connecting with a amplifying circuit, with ultrabroad range from the visible to FIR. The photodetector shows the ultrafast response time around 4 ns and energy resolution better than 1 THz.^[^ ^16^ ^]^ These two mechanisms show great development potential.

### 2.2 IR Imaging

On one hand, there are many information carried by the light in the macro‐ and microaspects. The linear propagation of light makes it reversible, from which we can get the position information of the object. And the outline and boundary of an object are presented by the contrast of light and shadow, which is originated from the different intensity of light on the edge of the object. In addition, from the microaspect, a beam of light carries all kinds of detectable information, such as the wavelength and the corresponding photon number, the polarization and corresponding vibration direction, the phase position and corresponding wave period. On the other hand, there are also information that can be obtained by detection. We can measure the photon energy and photon numbers by photoelectric effect and also the light intensity by photothermal effect. The polarization state of the light can be obtained by the anisotropic crystal. All the detectable information can be used for imaging by collected in a certain order. So far, two imaging schemes are widely used in the literature, including single pixel imaging based on the movement of the photodetector or object, and array imaging with the photodetectors placed on the focal plane of the light.

#### 2.2.1 Single Pixel Imaging

**Figure 3:**
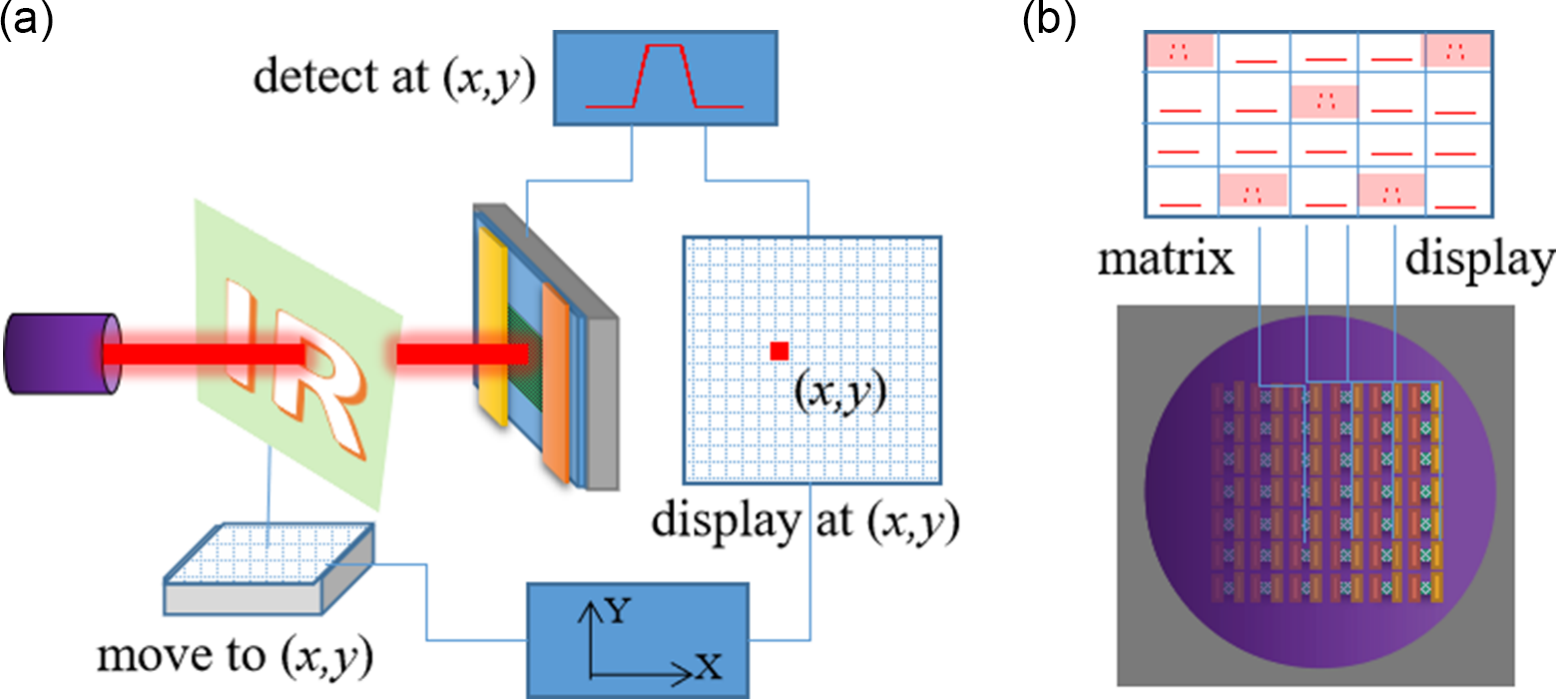
The mechanism of IR imaging by the a) single pixel mode and b) array mode. In the single pixel imaging, there is only one potodetector. The object with the letter “IR” should be moved slightly by a motion controller on the plane and the precise position information (*x,y*) is recorded to correspond to the detection signal as a single pixel. The image is formed by many pixels as the object moves. However, the array imaging is like a screen with the signal from each photodetector as a pixel.

Single pixel imaging is that in which the image is produced by the position moving of a single detector. The single pixel camera adopts structured light illumination at the illumination end and single pixel light intensity detector at the detection end to collect signals. When the lighting structure changes, the change of the corresponding object light intensity reflects the correlation between the lighting structure and the spatial information of the object. **Figure** **3**a shows a simple single pixel imaging mode commonly used in the laboratory. Fix the detector directly to the light source, place the shield with hollow “IR” between the light source and the detector, move the shield step by step through the console and record the position information (*x*, *y*) accurately. When the light source is blocked, the detector has no signal; and when the light source irradiates the detector through the hollow, the detector generates signal. The detection pattern can be formed by combining the signal of the detector with the position information of the baffle. The resolution of the detection pattern is related to the step size of the console. By changing the lighting structure and accumulating the correlation information, the object can be imaged. Since the single pixel camera only needs light intensity detection at the detection end, its requirements for the detector are far lower than those of the area array detector in ordinary imaging. Therefore, for some special conditions, the single pixel imaging technology has great application advantages. Because of this advantage, the research of single pixel camera has received great attention.

#### 2.2.2 Array Imaging

The other mode is to assemble a single detector into a detection array to collect light from different angles at the same time, as shown in Figure 3b. The intensity of the light signal received by a single component is used to form a gray image, so as to directly display the shape of the target. In the laboratory, a shielding board with a hollow pattern is usually placed parallel to the detection array panel and exposed with a light source. Thus, the blocked detectors have no signal and the detectors at the hollow position have signals, forming the outline of the hollow pattern. Furthermore, the ultimate goal of array imaging is to imitate the human eye by putting the detector array on the IR focal plane. The front part is the lens and other condensing devices, and the back end is connected with the logic processor to realize stereo imaging and automatic tracking imaging. At present, the array imaging device is still in the preliminary design stage, which can only realize the passive recognition of optical signals. Based on the complexity of manufacturing, it can be divided into linear array and area array. Linear array is a combination of detectors with simple layout and design. Now thousands of units long linear array can be produced for scanning imaging. The area array is not only a combination of linear array, but also involves crisscross wiring and signal control, which is extremely complex.^[^ ^17^ ^]^ The commercial 4 K × 4 K units structure can be used for staring imaging.^[^ ^18^ ^]^

## 3 2D Materials for IR Detection

2D materials have attracted many research topics due to their unique structure and excellent electronic and optical properties. So far, dozens of 2D materials have been reported for IR photoelectric detection and imaging. They can be divided into broadband type and special band type. Because the peak of the radiation corresponding to the ambient temperature is about 10 μm, the detection range of most reported photodetectors is as of 10 μm. And deep IR detection needs to be carried out at low temperature to eliminate interference. For example, the commercial HgCdTe detector in liquid nitrogen can measure the IR more than 20 μm, whereas the commercial 4 K combined calorimeter in liquid helium can measure the wavelength more than 100 μm in the FIR. Broadband detection imaging can be used for shape recognition. While special detection is for the different application. The MIR of 3–5 μm has special scientific significance for molecular fingerprint, remote sensing, and communication. The NIR has important technical significance for thermal imaging and night vision. Here, we introduce some promising material and their achievements as photodetectors, progressive designs of state‐of‐the‐art for the performance improvement, and also fresh physical mechanism for the photoelectronic detection (**Table** **1**).

**Table 1:** The 2D material for IR detection

Material		Broad *λ* [μm] (Special *λ*)	*R* [A W^−1^]	D* [Jones]	[*T*] *t*(rise)/*t*(fall)	Ref.
G‐like	G	300–30 (3.4 THz)	30 V W^−1^	10^10^	0.9/1.4 ns	[25]
	Te	0.52–3.39(1.55)	19.2 m	–	4.3 ns	[29]
		(3)	–	10^16^	280 ns	[30]
	BP	0.94	4.8 m	–	1/4 ms	[31]
	b‐PC	0.5–8(2.004)	2163	10^14^	0.7 ns	[34]
	b‐AsP	0.5–8.2(3‐5)	0.2–0.1	10^9^	0.5 ms	[35]
	BP‐InSe	0.45–1.55(1.55)	43.11	–	22 ms	[33]
VI B‐VI A	Cr_2_S_3_	0.52–1.55(1.55)	3	10^9^	1.7/1.65 s	[36]
	MoS_2_–G	0.4–2(2)	376	10^10^	0.6 s	[65]
	WS_2_–Si	0.2–3.043(0.98)	0.02	10^13^	4.5/21.7 ms	[69]
	MoTe_2_– (h‐BN)	(1.31)	0.1	10^8^	37.5 /50 μs	[70]
V–VI	Bi_2_Se_3_–Si	(2.7)	0.21	10^10^	23 ms	[39]
	Bi_2_Se_3_	0.33–1.55(1.456)	2.7	10^10^	0.5 s	[40]
	Bi_2_Te_3_	(1.55)	778	–	>1 s	[41]
	Bi_2_O_2_Se	(0.8)	6.5	10^11^	2.8 ms	[44]
		(1.55)IR‐ THz(0.17 THz)	58, 10^4^ V W^−1^	10^12^	476 ns	[45]
VIII–VI	PtSe_2_	(10)	4.5	10^8^	1.2 ms	[48]
	PtSe_2_–G	(10.6)	0.3	10^7^	50 ms	[49]
	PtTe_2_–Si	0.2–10.6(3–10.6)	5–0.67	10^9^	2.4 μs	[50]
	CoSe	0.45–10.6(10.6)	2.58	10^9^	73/80 ms	[51]
	Fe_3_O_4_	0.3–10.6(10.6)	561.2	10^8^	0.9/0.7 s	[52]
	PdSe_2_	0.2–4.6(4.6)	0.726	10^14^	3.5/3.9 μs	[78]
	PtTe_2_	0.2–1.65(0.98)	0.406	10^12^	7.5/ 36.7 μs	[82]

### 3.1 G‐Like Materials for Broadband

Since the discovery of G in 2004, the excellent photoelectric and mechanical properties have attracted a worldwide research and development boom onto ultrathin nanomaterials. G has excellent electron transport properties and the ability to absorb similar to 2% of incident light over a broad wavelength range.^[^ ^19^ ^]^ And it has resonant response to photons in the ultrawideband spectrum range of UV to THz wave with the zero bandgap electronic structure. Kawano^[^ ^20^ ^]^ reported that the graphene‐based transistor is capable of detecting THz and IR waves (0.76–33 THz), with the frequency‐selective under a tuned magnetic field. Trung et al.^[^ ^21^ ^]^ reported a kind of transparent and flexible IR photodetector using a field‐effect transistors (FETs) structure with the layered r‐GO and poly as the channel. The photodetector exhibits high IR responsivity, stability, and reproducibility under mechanical strain and ambient conditions. And the capability of measuring the distribution of responses from each device in the transparent and flexible nanocomposite FET array under IR radiation from the human body is also demonstrated. Sun et al.^[^ ^22^ ^]^ reported an efficient MIR room‐temperature photodetector enhanced by plasmonic effect in graphene nanoresonators (GNRs)‐G heterostructure. The plasmon polaritons in GNRs are size dependent with strong field localization. The performance of the photodetector can be enhanced by incorporating GNRs to enhance the light absorption. Sun and Chang^[^ ^23^ ^]^ reported the review of mechanisms and methodology in photodetection via G and G‐like 2D materials, which have broad application prospects in the fields of electronics and optoelectronics. Prechtel et al.^[^ ^24^ ^]^ reported that built‐in electric fields give rise to a photocurrent to 4 ps and that the photothermoelectric effect generates a current with a decay time to 130 ps at the G‐metal interface by the time‐resolved picosecond photocurrents measurement techniques. Viti et al.^[^ ^25^ ^]^ report room‐temperature THz nanoreceivers exploiting antenna‐coupled G FET, operating with a combination of high response time (hundreds of ps) and high sensitivity (NEP below 120 pW Hz^−1–2^ at 3.4 THz).

Wang et al.^[^ ^26^ ^]^ reviewed the group of 2D single‐element nanomaterials that called 2D‐Xenes, which used to make photodetectors with high responsivity, broad spectral response range, fast response speed, and high specific detectivity. For example, Hu et al.^[^ ^27^ ^]^ reported an ultrathin nonlayered Ge flakes via halide‐assisted self‐limited chemical vapor deposition (CVD) growth for high‐performance phototransistors. Xiao et al.^[^ ^28^ ^]^ reported an antimonene‐based flexible photodetector, which showed a responsivity of 10 μA W^−1^ and on–off ratio of 26.8 under the bias of 1 V. Shen et al.^[^ ^29^ ^]^ report air‐stable 2D Te nanoflakes for broadband and ultrasensitive photodetection via hydrothermal synthesized, with the peak extrinsic responsivity of 383 A W^−1^, 19.2 mA W^−1^, and 18.9 mA W^−1^ at the illumination of 520 nm, 1.55 μm, and 3.39 μm, respectively. In addition, the photodetector exhibits an exceptionally high anisotropic behavior at the communication wavelength of 1.55 μm with a large bandwidth of 37 MHz. Deckoff‐Jones et al.^[^ ^30^ ^]^ reported an atomic thin crystal of element Te, which is a multifunctional material and suitable for MIR optoelectronics. They design an waveguide‐integrated Te photodetector and pockels effect modulator, with the room temperature NEP is as low as 0.03 fw Hz^−1/2^ at 3 μm. Buscema et al.^[^ ^31^ ^]^ studied the photoresponse of FETs made of few‐layer black phosphorus (BP), showing the response to excitation wavelengths from the visible region up to 940 nm and a rise time of about 1 ms with responsivity reaches 4.8 mA W^−1^. Viti et al.^[^ ^32^ ^]^ devised the first room‐temperature terahertz (THz)‐wave nanodetector by a 10 nm‐thick BP as an active channel. Cao et al.^[^ ^33^ ^]^devised a photogate vertical structure by vertically stacking layered InSe on top of layered BP, possessing a wide response range from 405 to 1550 nm, with a relatively fast response (22 ms) and high responsivity to 53.80 A W^−1^ at 655 nm and 43.11 A W^−1^ at 1550 nm, respectively. Recently, Tan et al.^[^ ^34^ ^]^ reported a new black phosphorous carbide (b‐PC) with absorption spectrum up to 8000 nm. At 2004 nm, the peak responsivity of the b‐PC phototransistor reaches 2163 A W^−1^ and the NEP of 1.3 fW Hz^−1–2^, with the response time of 0.7 ns adjusted by gating effect. Long et al.^[^ ^35^ ^]^ reported the black arsenic phosphorus (b‐AsP) for room temperature high‐detectivity MIR photodetectors, which works up to 8.2 μm with the detectability beyond 10^9^ Jones in the range of 3–5 μm with the zero bias, realizing fast light response and low dark noise.

To sum up, G‐like 2D materials generally have small bandgap and wide detection spectrum. Due to the zero bandgap and excellent electron transport properties, G has ultrawide‐band spectrum range of UV to THz wave with ultrafast response speed up to ps. 2D Te is especially suitable for MIR optoelectronics with high anisotropic behavior at the communication wavelength of 1.55 μm and the room temperature NEP as low as 0.03 fw Hz^−1/2^ at 3 μm. In addition, 2D BP, b‐PC, and b‐AsP are all good detector in MIR with high‐detectivity and fast response time.

### 3.2 Group VI B–VI a for NIR

Similar to G while with bandgap, Group VI B–VI A materials are another kind of important 2D materials, which include MoS_2_, MoTe_2_, WS_2_, WSe_2_, etc. As semiconductor with the bandgap of about 1 eV, they have high responsivity and detectivity for NIR. The mobility of single‐layer transition metal disulfides (TMDs) is about 10^2^ cm^2^ V^−1 ^s^−1^ at room temperature, which leads to fast response speed. In addition, Xie et al.^[^ ^36^ ^]^ reported a new VI B–VI A semiconductor of air‐stable narrow‐gap Cr_2_S_3_ for broadband photodetection with thickness less than 1.85 nm grown by CVD, which has excellent environmental stability that its degradation can be ignored for 2 months. The Cr_2_S_3_ nanosheets with bandgap of 0.15 eV show high responsivity (14.4 A W^−1^ at 520 nm, 6.0 A W^−1^ at 808 nm, and 3.0 A W^−1^ at 1550 nm) and excellent detection rate (4.0 × 10^10^ Jones at 520 nm, 1.7 × 10^10^ Jones at 808 nm, and 8.3 × 10^9^ Jones at 1550 nm) under environmental conditions.

Group VI B–VI A 2D materials are good semiconductors with high single‐layer mobility and good environmental stability. Their bandgap decided that they can be made as NIR detectors. The new narrow‐gap Cr_2_S_3_ may give some surprises.

### 3.3 Group V–VI for NIR and MIR

V–VI semiconductors are also 2D topological insulators (TIs).^[^ ^37^ ^]^ Among the available materials, TIs has a high sensitivity and wide spectrum detection ability, with a high carrier mobility and good environmental stability. Huang et al.^[^ ^38^ ^]^ fabricated a robust photodetector by Bi_2_S_3_ nanosheet films, with the high responsivity at a lower bias potentials and strong long‐term stability of the switching behavior without any external protection in alkaline solution. Luo et al.^[^ ^39^ ^]^ reported a narrow bandgap (0.3 eV) of layered vertical Bi_2_Se_3_ for MIR photodetection. The as‐prepared vertical Bi_2_Se_3_‐Si heterojunction shows excellent photoresponse (photo‐to‐dark ratio of 2.0 × 10^4^) and extremely low dark current (0.21 pA), with a response time of 23 ms and a specific detectivity of 1.2 × 10^10^ Jones under 2.7 μm illumination at room temperature. In addition, Wang et al.^[^ ^40^ ^]^ reported large size (0.2–0.4 mm) and ultrathin (3 nm to few nanometers) 2D Bi_2_Se_3_ flakes with high crystal quality, which exhibits an ultrahigh on–off current ratio of 10^6^ and responsivity of 23.8 A W^−1^, with external quantum efficiency of 2035% at 1456 nm in the E‐band of the telecommunication range. Sharma et al.^[^ ^41^ ^]^ reported robust photoconductivity measurements on Bi_2_Te_3_ nanosheets and nanowires from UV to NIR, with the ultrahigh responsivity (to 74 A W^−1^ at 1.55 μm), without affecting the performance even after 4 months of storage at ambient conditions. Yao et al.^[^ ^42^ ^]^ reported an all‐layered 2D Bi_2_Te_3_–SnSe–Bi_2_Te_3_ photodetector. The broadband photoresponse of the device is from UV to NIR and the optimized responsivity reaches 5.5 A W^−1^, with the corresponding eternal quantum efficiency of 1833% and detectivity of 10^10^ Jones at 808 nm.

Chitara et al.^[^ ^43^ ^]^ reported an ultrathin Bi_2_O_2_S nanosheet (2–3 nm) for NIR photodetectors, with high performance, under 785 nm laser illumination. The device shows a responsivity of 4 A W^−1^, an external quantum efficiency of 630%, and a normalized photo‐to‐dark current ratio of 1.3 × 10^10^ per watt with a fast response time of 100 ms. Li et al.^[^ ^44^ ^]^ reported the ultrathin Bi_2_O_2_Se nanosheets (with the thickness below 10 nm and lateral sizes about 10 μm) via a low‐pressure chemical vapor deposition method. The NIR photodetector shows a response time, responsivity, and detectivity up to 2.8 ms, 6.5 A W^−1^, and 8.3 × 10^11^Jones, respectively, without changing from 80 to 300 K due to less surface trap states and shallow defect energy levels. In addition, Chen et al.^[^ ^45^ ^]^ reported an antenna‐assisted Bi_2_O_2_Se photodetectors with a broadband spectra from IR to terahertz, which driven by multimechanism of electromagnetic waves to electrical conversion. The photodetector achieves a broadband responsivity of 58 A W^−1^ at 1550 nm, 2.7 × 10^4^ V W^−1^ at 0.17 THz, and 1.9 × 10^8^ V W^−1^ at 0.029 THz, respectively, with an ultrafast response time of 476 ns and a quite low NEP of 0.2 pW Hz^−1–2^ at room temperature. Fu et al.^[^ ^46^ ^]^ reported an ultrasensitive 2D phototransistor using CVD‐grown 2D Bi_2_O_2_Se transferred onto silicon substrates. The phototransistors show a responsivity of 3.5 × 10^4^ A W^−1^, a photoconductive gain of more than 10^4^, a time response in the order of sub‐ms, and an ultrahigh sensitivity with a specific detectivity of 9.0 × 10^13^ Jones. Khan et al.^[^ ^47^ ^]^ reported an ambient‐pressure vapor–solid (VS) deposition approach for the growth of millimeter‐sized 2D Bi_2_O_2_Se single crystal, with thicknesses down to monolayer and good crystalline quality, chemical uniformity, and stoichiometry. The phototransistor exhibits a maximum responsivity beyond 10^4^ A W^−1^, and photodetectivity up to 10^15^ Jones with gate tunable.

As the 2D TIs, Group V–VI semiconductors usually have high carrier mobility and good environmental stability. The narrow bandgap makes them suitable for MIR detection with high external quantum efficiency. The new material Bi_2_O_2_Se maybe can create magic as it has an ultrahigh responsivity beyond 10^4^ A W^−1^ and special detectivity up to 10^15^ Jones with broad spectra from IR to terahertz.

### 3.4 Group VIII–VI for Broadband

Recently, Group VIII–VI have been developed a series of materials for excellent MIR and broadband spectra detection. Yu et al.^[^ ^48^ ^]^ successfully synthesized narrow bandgap 2D PtSe_2_ crystal with large area. Experiments show the single‐layer PtSe_2_ FET devices are suitable for visible and NIR photodetectors, and double‐layer PtSe_2_ FET devices are suitable for broadband MIR photodetectors, in which the bandgap can be further modulated by defect engineering. Long et al.^[^ ^49^ ^]^ report a method of developing a scalable room temperature operating device array with PtSe_2_‐G heterojunction, with the responsivity of 0.3 A W^−1^ at 10.6 μm in the MIR region. Zeng et al.^[^ ^50^ ^]^ reported the growth of a wafer‐scale 2D PtTe_2_ layer via a simple tellurium‐vapor transformation approach. The PtTe_2_–Si Schottky junction photodetector show an ultrabroadband light of up to 10.6 μm with a high specific detectivity, with the room‐temperature IR‐imaging capability.

Liang et al.^[^ ^51^ ^]^ studied the photodetector based on air‐stable 2D ferromagnetic material CoSe at room temperature, with the photoresponse ranges from 450 nm to 10.6 μm. The photoresponsivity is up to 2.58 A W^−1^ under the 10.6 μm illumination at room temperature. Yin et al.^[^ ^52^ ^]^ introduced a high performance and ultrawideband photodetector from UV to MIR based on space constrained CVD synthesis of air‐stabilized nonlayered Fe_3_O_4_ nanosheets. At the wavelength of 10.6 μm, 561.2 A W^−1^ ultrahigh photoresponse (*R*), 6.6 × 10^3^% external quantum efficiency (EQE), and 7.42 × 10^8^ Jones special detectivity (D*) were obtained. The multi mechanism synergy of photoconductive effect and radiative heat effect proves the high sensitivity to any light intensity.

Group VIII–VI 2D materials, such as PtSe_2_, PtTe_2_, CoSe, and Fe_3_O_4_ nanosheets, are novel narrow gap semiconductors with photoresponse ability up to 10.6 μm at room temperature. They are promising to open the door of all infrared world.

In addition, the weak van der Waals interaction in 2D materials inspired researchers to explore high responsivity and detectivity 2D heterostructure broadband photodetectors in the MIR region. Attributed to the atomically thin and intrinsic band structure, 2D materials photodetectors often show an inevitable compromise between photodetectivity and photoresponsivity with one high and the other low. An effective solution is to construct heterojunctions, which will produce ultralow dark currents resulting from the depletion region at the junction and high direct tunneling current when illuminated.^[^ ^53^ ^]^ Based the matching bandgaps, the heterostructures tend to apply in NIR to FIR regions for high responsivity and detectivity. Visible‐to‐NIR photodetectors have been reported based on MoS_2_ vertical Schottky junctions.^[^ ^54^ ^]^ A highly sensitive of WSe_2_–SnS_2_ vdW heterostructure photodiode is constructed on BN thin film by exfoliating each material and manually stacking them, showing both ultrahigh photodetectivity of 10^13^ Jones and photoresponsivity of 244 A W^−1^ at a reverse bias under the illumination of 550 nm light.^[^ ^55^ ^]^ The G–S‐doped InSe heterostructure photodetectors with excellent photoresponse performance achieve an ultrahigh photoresponsivity of similar to 4.9 × 10^6 ^A W^−1^ at 700 nm and an EQE of 8.7 × 10^8^% with visible‐to‐near‐IR photodetection.^[^ ^56^ ^]^ Based on the epitaxial heterostructures of WS_2_–G, highly responsive and broadband photodetectors are built.^[^ ^57^ ^]^ Upon light illumination, photoexcited carriers are separated by the built‐in field at the WSe_2_–WS_2_ heterojunctions, with holes trapped in the WSe_2_ nanodots.^[^ ^58^ ^]^ Others such as p–n heterojunction of BP–MoS_2_,^[^ ^59^ ^]^ AsP–InSe,^[^ ^60^ ^]^ and Schottky junction of G‐InAs,^[^ ^61^ ^]^ G‐Bi_2_Te_3_,^[^ ^62^ ^]^ and G‐MoS_2_,^[^ ^63^ ^]^ are all designed for the enhanced responsivity and detectivity of the devices. The band structure engineering in MoS_2_‐based heterostructures toward high‐performance phototransistors is also reviewed.^[^ ^64^ ^]^ A G‐MoS_2_‐G vertical heterostructure is demonstrated. The photodetector shows excellent performance with an enhanced responsivity of 376 A W^−1^ at 2000 nm, and a broad working wavelength ranging from 405 to 2000 nm.^[^ ^65^ ^]^ Another novel 2D h‐BN‐p‐MoTe_2_‐G‐n‐SnS_2_‐h‐BN p–g–n junction, fabricated by a layer‐by‐layer dry transfer, demonstrates high‐sensitivity, broadband photodetection at room temperature. An optimized device containing 5–7‐layer G has been achieved and shows an extraordinary responsivity exceeding 2600 A W^−1^ with fast photoresponse and specific detectivity up to approximate to 10^13^ Jones in the NIR spectrum.^[^ ^66^ ^]^

## 4 Device for IR Imaging

IR imaging has gained intensive attention due to its various applications, including night vision, remote sensing, medical imaging, industry defect imaging, etc. The 2D materials now are used to solve the traditional problem in IR imaging by making use of advances in nanofabrication and nanotechnology.^[^ ^67^ ^]^ According to the principle of the IR imaging, it can be divided into single pixel mode and array mode. depended on the difference of detection index, the obtained image contains two kinds of types, imaging based on intensity and imaging based on polarization. Thus, four combinations are used to describe the IR imaging based on 2D materials, that is, single pixel imaging based on intensity (SI), single pixel imaging based on polarization (SP), array imaging based on intensity (AI), and array imaging based on polarization (AP). The 2D material‐based IR imaging is shown in **Table** **2**.

**Table 2:** IR imaging based on 2D materials

Material	*λ* [μm]	Mode	Special parameter	Others
BP	0.83	SI	64 × 64 pixel via 2000 tests	
WS_2_–Si	0.98	SI	zero bias	D* to 10^13^ Jones
MoTe_2_–(b‐BN)	1.31	SI	Weak dependence at 80–300 K	Response time 37.5/50 μs
PtTe_2_–Si	3.04–10.6	SI,AI	Fast speed ≈2.4 μs	3–60 layers
Te	1.55, 2.3	SP	DoLP to 0.8	
PdSe_2_–Si	0.98, 4.6	SP	D* to 10^14^ Jones	DoLP to 0.75
CsPbBr_3_–Mxene	Array	AI	1665 pixel at 72 cm^2^	
PtTe_2_	0.98	AI	8 × 8 array	large area films 4.81–96.7 nm

### 4.1 Imaging Based on Intensity

Miao et al.^[^ ^68^ ^]^ design a single pixel photodetector for NIR imaging based on BP using compressed sensing algorithm, which can overcome the difficulties of BP film growth and the fabrication of scalable photodetector array. The original IR image can be decoded and reconstructed from the current signal collected by the BP photodetector. IR laser spot images of 32 × 32 and 64 × 64 pixels are obtained successfully via 500 and 2000 measurements, which is only half of the total number of pixels. Wang et al.^[^ ^69^ ^]^ reported a hybrid dimensional heterojunction fabricated by 2D WS_2_‐3D Si for high‐performance IR photodetection and imaging applications. The device has a broadband response range of 200–3043 nm. Under 980 nm illumination, a large responsivity of 20 mA W^−1^, a high special detectivity of 4.3 × 10^13^ Jones and a fast response speed of 4.5–21.7 ms were obtained under zero bias. It is worth mentioning that the device also has excellent IR imaging ability and high image resolution based on the SI mode. Tong et al.^[^ ^70^ ^]^ applied the tunneling mechanism to the optical detection of the MoTe_2_‐(*h*‐BN)‐MoTe_2_‐(*h*‐BN) heterostructure at 1310 nm. Under the reverse leakage bias, the tunneling behavior is mainly controlled by holes to ensure the high light responsivity and the dependence on temperature is weak. A fast light response speed, with a rise time of about 37.5 μs and a fall time of 50.0 μs, is observed and device is feasible for practical imaging applications by the SI mode.

Zeng et al.^[^ ^50^ ^]^ realized the high‐quality 2D‐PtTe_2_ layer on Si synthesized at relatively low temperature (≈450 °C) by Te vapor conversion method. The number of layers can be reasonably adjusted from 3 to 60 by precisely adjusting the thickness of the initial Pt film. Due to the few interface defects in VDW epitaxial 2D layer, the PtTe_2_–Si photodetector shows high sensitivity in the ultrawideband spectral range of 0.2–10.6 μm, with a fast response speed of ≈2.4 μs and a room temperature ratio detection rate of 0.93–6.92 × 10^9^ Jones at 3.04–10.6 μm. The high resolution IR image sensor based on SI and AI modes all has good imaging performance at room temperature, indicating great potential for MIR photodetectors and imaging.

### 4.2 Imaging Based on Polarization

Polarized light signal can be used for polarization detection and imaging. As a new technology, IR polarization detection has a broad application prospect in the military and civil fields.^[^ ^71^ ^]^ Due to the polarization characteristics determined by the object in the process of reflection and radiation, the amplitude of the vertical and parallel components of the electric vector in the reflected light changes, bringing the partially polarized reflected light. Different objects or different states of the same object (such as surface, physical, and chemical properties, etc.) often have different polarization states in the IR waves. Using IR polarization imaging technology, multidimensional feature information such as intensity, polarization, and image of target can be obtained comprehensively, which can effectively improve the contrast between target and background, highlight the detailed features and enhance the effect of recognition. In addition, there are obvious differences in IR polarization characteristics between natural objects and man‐made objects, which can constitute new information for target detection.^[^ ^72^ ^]^ The emerging 2D materials, with high polarization photosensitivity and strong quantum confinement due to the anisotropic crystal structure, are easy to integrate on complex structures, which will meet the requirements of miniaturization, and flexibility in the next‐generation photodetectors.^[^ ^73^ ^]^ To far, there are many 2D materials to be used for the polarized photodetection. Tian and Liu^[^ ^74^ ^]^ reported a van der Waals heterojunction ReSe_2_–WSe_2_ photodetector, with a high responsivity of 0.28 A W^−1^ and a high detectivity of 10^12^ Jones under the illumination of 520 nm at room temperature. In addition, scanning photocurrent mapping (SPCM) measurements demonstrate the photoresponse of devices closely depend on the polarized angle of the incident light, indicating the effective polarized light detection. Zhao et al.^[^ ^75^ ^]^ reported a vertical p–n diode by vertically stacking p‐type few‐layer black phosphorus (BP) on n‐type few‐layer indium selenide (InSe). The device demonstrates a highly polarization‐sensitive photocurrent with an anisotropy ratio of 0.83 at a zero‐bias photovoltaic mode, a fast photoresponse and low dark current. Zhong et al.^[^ ^76^ ^]^ demonstrated the strong linear dichroism behavior of PdSe_2_ by polarization‐resolved absorption spectra measurements. The photodetector based on 5 L PdSe_2_ exhibited a significant photocurrent on–off ratio (10^2^), the quite fast response time (11 ms), and robust linearly dichroic ratios approximate to 1.9.

**Figure 4:**
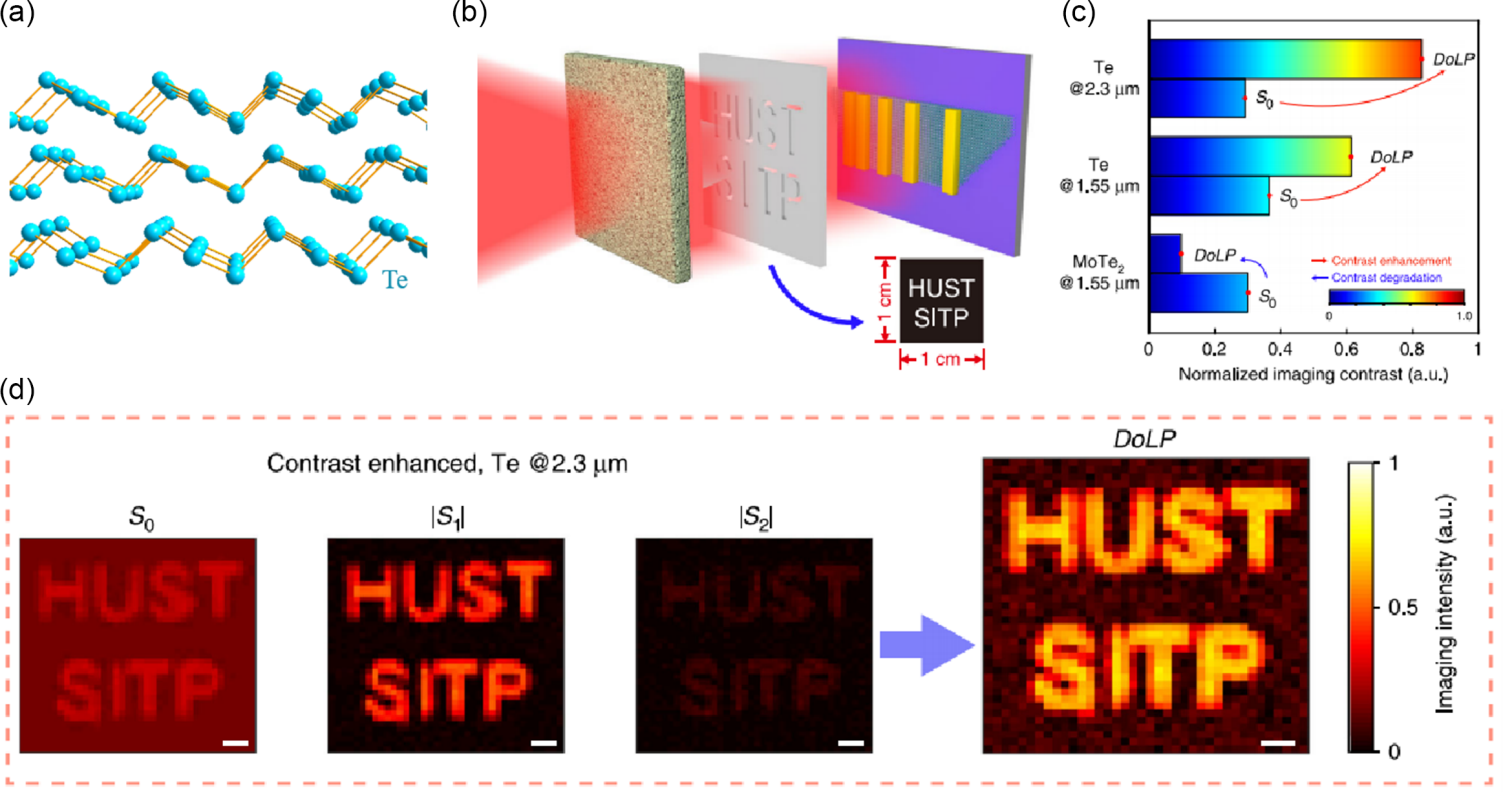
The polarization imaging system for IR detection with the 2D Te. a) The structure of 2D Te. b) Test system for the polarization detection and imaging. c) The comparison of polarization detection capability by Te and MoTe_2_ at 1.5  and 2.3 μm. d) The result of the polarization imaging.b–d) Reproduced under the terms of the CC‐BY 4.0 license.^[^ ^77^ ^]^ Copyright 2020, The Authors, published by Springer Nature.

Tong et al.^[^ ^77^ ^]^ introduced the anisotropic Te photodetector as the polarized IR imaging for a designed target obscured by scattering media at room temperature under 2.3 μm illumination, with excellent stability. As shown in **Figure** **4**, the optical response anisotropy ratio of the photodetector is as high as 0.8 under 2.3 μm illumination. As a new star in 2D material field, Te‐based photodetectors have broad application prospects in the next generation of imaging equipment after overcoming the challenge of controllable growth of large‐scale film. Wu et al.^[^ ^78^ ^]^ point out that mixed‐dimensional PdSe_2_–Si heterostructure‐based photovoltaic detectors can used for self‐driven, broadband photodetection, IR imaging, and humidity sensing. The device has a high sensitivity to the ultrawideband spectrum of 0.2–4.6 μm, with a responsivity of 726 mA W^−1^, a large special detectivity of 3.19 × 10^14^ Jones, a response speed of 3.5 ms, and a polarization sensitivity of 0.75. It has excellent imaging capability under 0.98 and 4.6 μm illumination based on SP mode, which show that the great potential in high‐performance polarization sensitive and IR imaging.

### 4.3 Integration Array

**Figure 5:**
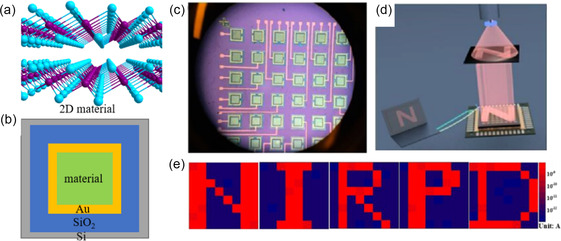
A typical array imaging system for IR detection. a) The 2D material, b) the photodetector, c) the array fabricated on the substrate, d) the test system for IR imaging and e) the result are shown. c–e) Reproduced with permission.^[^ ^82^ ^]^ Copyright 2020, American Chemical Society.

Using semiconductor technology, the 2D material‐based photodetector can be integrated with logic devices and display devices to realize the intellectualization. It can form a huge application prospect with a great development in the field of artificial intelligence. The integrated multifunctional full spectral stereo detector has shown its powerful visual simulation ability, and many of its performance can far surpass the human eye and serve various signal collection fields. For the realization of digital twin and virtual reality, more powerful photodetectors are needed to collect and feedback various kinds of physical signals. Conventional semiconductors such as Si‐ and InGaAs‐based photodetectors have complementary metal oxide‐semiconductor (CMOS) incompatibility. While the 2D materials can circumvent all these issues benefiting from mechanically flexibility. Due to the van der Waals interaction between 2D materials and Si, the formation of heterostructures is not limited by lattice mismatch. Consequently, the integration process can be achieved easily. G and other 2D material‐based optoelectronic capabilities can augment CMOS devices for high‐speed and low‐power optical interconnects. Shiue et al.^[^ ^79^ ^]^ demonstrated an on‐chip ultrafast photodetector based on a 2D heterostructure consisting of high‐quality G encapsulated in h‐BN. Coupled to the optical mode of a silicon waveguide, the device exhibits a maximum responsivity of 0.36 A W^−1^ and high‐speed operation with a 3 dB cutoff at 42 GHz by hot electron‐mediated effects (**Figure** **5**).

In addition, the flexible integration of photodetectors beyond CMOS are attracting more attention due to their promising applications in wearable optoelectronic devices, bendable imaging sensors, and implantable optoelectronics. Deng et al.^[^ ^80^ ^]^ demonstrate an all‐sprayed‐processable and large‐area photodetectors on common paper based on 2D CsPbBr_3_ nanosheets and conductive Ti_3_C_2_T_x_ (MXene) for a 1665 pixel array in 72 cm^2^. The device exhibit an on‐off current ratio up to 2.3 × 10^3^ and photoresponse speed to 18 ms, with the special detectivity of 6.4 × 10^8^ Jones and responsivity of 44.9 mA W^−1^ under a bias of 10 V. Moreover, the device can still maintain the excellent flexibility and stability after bending 1500 cycles. Goossens et al.^[^ ^81^ ^]^ report the monolithic integration circuit with G, operating as a high‐mobility phototransistor with the sensitive wavelength at 300–2000 nm. The integration is pivotal for incorporating 2D materials into the next‐generation microelectronics, sensor arrays, low‐power integrated photonics and imaging systems covering visible, IR, and terahertz frequencies.

Tong et al.^[^ ^82^ ^]^ reported the large‐area PtTe_2_ films prepared directly from PtTe_2_ nanofilms with different thicknesses ranging from 4.81 to 96.7 nm. The photodetector displays a wide‐band optical response in the range of 200–1650 nm, with the responsivity and special detectivity of 0.406 A W^−1^ and 3.62 × 10^12^ Jones, respectively. And the response time of rise and fall is 7.5 and 36.7 μs, respectively. The image sensor composed of 8 × 8 array can record NIR images below 980 nm based on AI mode with satisfactory resolution. Lan et al.^[^ ^83^ ^]^ review the recent progress of semiconducting 2D materials in IR optoelectronic devices, with the background and the suitable materials for IR applications. Due to the van der Waals interaction between 2D materials and Si, the lattice mismatch of 2D materials and Si can be neglected; consequently, the integration process can be achieved easily.

## 5 Conclusion

In this review, we focus on the IR detection and imaging based on 2D material. The IR radiation is originated from the higher energy level to lower energy level of electrons, atoms or molecules, transmitting as a photon with a specific frequency. However, the IR detection is a opposite process. The IR photons are absorbed by material and causes energy changes of electrons, atoms, or molecules in the material. Then the varying electrical parameters is convenient to be observed. According to what we observe, the detectable information is obtained, and the imaging is the sequence of the information. Among the aforementioned, the material plays a key role in the detection and imaging. With the serious manufacturing cost and integration problems, Si‐based photodetectors are limited to small batch markets, and have no chance to enter the autopilot and industrial vision field based on IR detection and imaging. The 2D materials, with thousands of species and high performance in various devices, provides a broad prospect to the challenge. Research shows that the 2D materials‐based photodetectors for IR detection can reach a broad wavelength up to 10 μm, with the responsivity and special detectivity to 10^8 ^A W^−1^ and 10^15^ Jones, respectively.

We main discusses the material selection and the device design for IR imaging in the review. So far, dozens of 2D materials have been reported for IR detection and imaging. They are simply divided into four categories, the G‐like materials are a group of 2D single‐element nanomaterials and their derivation. They usually have the broadband response. The G and BP have been widely studied with their excellent properties. It is worth noting that some new materials like Te, b‐PC, and b‐AsP demonstrate the unexpected performance, which may cause the upsurge in research. The Group VI B–VI A materials include MoS_2_, WS_2,_ etc. They have always been the hotspot in 2D materials. The Group V–VI materials provide new elements for 2D family. They are always 2D TIs with the peculiar electrical properties. And Bi–O‐based materials show strong stability in air, with good responsivity and detectivity for NIR. The Group VIII–VI is a novel family. They exhibit the excellent detectivity for MIR. The materials of CoSe, PtSe_2_, PtTe_2_, and 2D Fe_3_O_4_ are all worth studying deeply. The IR imaging based on Group VIII–VI materials shows the omnipotence, whatever it is based on intensity or polarization. They may become a new star in the IR detection and imaging.

After more than 10 years of development, there have been thousands of research achievements in 2D material‐based photodetectors. Especially in the past 5 years, as a hot field, there have been a variety of novel research papers. As a result, a large number of new mechanisms and structures have been found, and many new problems have been derived. 2D materials are unnecessity of high‐vacuum materials growth technique, and are suitable for seamless integration in various substrates (rigid or flexible, single crystal or amorphous), so as to realize the integration of high‐performance optoelectronic devices with ultralow weight, high density, and flexibility. In addition, with high polarization sensitivity and strong quantum confinement due to the anisotropic crystal structure, the emerging 2D materials are easy to integrate on complex structures, which will meet the requirements of miniaturization, and flexibility in the next generation photodetectors. In the next 5 years, this field will always be a research hotspot and produce a large number of achievements. For application, the development of polarization, single pixel and integrated detection imaging has a long way to go. More importantly, a complete IR world contains a huge amount of valuable information. Due to the never‐ending thermal motion of molecules, all objects radiate IR waves all the time. It ignores the boundary between day and night. In the IR vision, the world is always bright as day, and the shape and outline of objects are clearly visible. Moreover, the IR spectra of different objects and different parts are different, which is easy to lock and track. For example, the IR imaging of human body can be used for medical exploration and etiology confirmation. In addition, IR spectrum comes from molecular vibration, which corresponds to molecular structure information. We can use IR spectrum mapping to study molecular chemistry and biochemistry. To realize these perspectives, a variety of high‐precision IR detectors are needed, which are depended on the development of material science. In brief, the achievements and challenges of 2D material‐based photodetectors for IR imaging will exist side by side in the future.

## Conflict of Interest

The authors declare no conflict of interest.
